# Physical Unclonable Function and Hashing Are All You Need to Mutually Authenticate IoT Devices

**DOI:** 10.3390/s20164361

**Published:** 2020-08-05

**Authors:** Ahmed Mostafa, Suk Jin Lee, Yesem Kurt Peker

**Affiliations:** TSYS School of Computer Science, Columbus State University, Columbus, GA 31907, USA; mostafa_ahmed@columbusstate.edu

**Keywords:** physical unclonable functions, arbiter, HMAC, SRAM, IoT device authentication, invasive attack

## Abstract

Internet of Things (IoT) has become the driving force in modern day technology with an increasing and rapid urge to create an intelligent, efficient, and connected world. IoT is used in manufacturing, agriculture, transportation, education, healthcare and many other business environments as well as home automation. Authentication for IoT devices is essential because many of these devices establish communication with servers through public networks. A rigorous lightweight device authentication scheme is needed to secure its physical hardware from cloning or side-channel attacks and accommodate the limited storage and computational power of IoT devices in an efficient manner. In this paper, we introduce a lightweight mutual two-factor authentication mechanism where an IoT device and the server authenticate each other. The proposed mechanism exploits Physical Unclonable Functions (PUFs) and a hashing algorithm with the purpose of achieving a secure authentication and session key agreement between the IoT device and the server. We conduct a type of formal analysis to validate the protocol’s security. We also validate that the proposed authentication mechanism is secure against different types of attack scenarios and highly efficient in terms of memory storage, server capacity, and energy consumption with its low complexity cost and low communication overhead. In this sense, the proposed authentication mechanism is very appealing and suitable for resource-constrained and security-critical environments.

## 1. Introduction

An Internet of Things (IoT) device can be considered as a form of embedded system that is limited in its memory capacity, processing power, and energy sources, i.e., batteries [1]. Traditional cryptographic key storing and authentication methods, such as using public key or symmetric key algorithms in IoT authentication, are not suitable for power-constrained IoT environments as they require too much memory, power, and high battery use [2]. Although securing the Internet and network have been the main focus of digital security for decades, IoT devices are still vulnerable to malicious cyberattacks, not only from a software perspective with privacy, user profiling, tracking, and authentication, but also to physical attacks [3]. With an increasing number of IoT devices being used in an array of industries, including healthcare, public frameworks, and smart cities, the need for more enhanced security methods is paramount in these environments. Most security techniques are vulnerable because of potential threats by data hackers. The tools and techniques that are used to ensure the information and systems security should always be up to date and utilized to their optimum performance. In traditional authentication methods where the device that stores the key is kept physically secure, cloning or side-channel attacks are not much of a concern. The situation is different for IoT devices because they need to be placed in various places including public places where attackers have the opportunity to tamper with the physical device. For example, the stored keys in the non-volatile memory can be read and used to maliciously initiate an attack. Thus, IoT devices require more rigorous and lightweight authentication schemes to prevent them from being tampered with physically [1,3,4]. Security researchers have recently paid more attention to PUFs in the security context because Physical Unclonable Functions (PUFs) eliminate the need to store keys in the nodes. PUFs are the outcome of a manufacturing process for Integrated Circuits (IC) where the physical variations in the microstructure are random and allow the creation of a function that is physically unclonable and unpredictable [3]. PUFs are proving to be an ideal solution to more secure mutual authentication schemes for IoT systems.

In this paper, we propose a two-factor mutual authentication mechanism between the IoT device and the server that it communicates with, based on deploying two PUFs in the IoT device along with a hashing algorithm. To eliminate the vulnerability introduced by storing a cryptographic key in the IoT device’s non-volatile memory [5], we utilize a SRAM (Static random-access memory) PUF in our proposed mechanism. We distill a cryptographic key from the SRAM PUF. We also use an Arbiter PUF to achieve a reliable two-factor mutual authentication. The proposed mechanism does not use encryption during the authentication process hence no encryption algorithm is required in the IoT device or the server for authentication. Using encryption algorithms dissipates relatively a large amount of energy in IoT devices [2], causing the device battery to drain more quickly. The key feature of the proposed authentication mechanism is only to utilize a hashing algorithm for the mutual authentication in comparison to the existing ones. That makes our mutual authentication mechanism lightweight and more desirable for power-constrained IoT network applications. Many conventional mutual authentication algorithms are vulnerable to physical attacks as they have to store a secret cryptographic key in the IoT device’s non-volatile memory [6,7,8] to be used in the encryption/decryption process. Our proposed authentication mechanism does not require storing the secret cryptographic key or any sensitive data in the IoT device memory, making it resilient to physical attacks.

Our contribution in this paper is a mutual authentication mechanism that provides:

- A novel two-factor mutual authentication approach for IoT devices in which a hashing algorithm is utilized for the IoT device and the server to authenticate each other,
- Use of two PUFs in the IoT device for two-factor authentication for stronger security against cyberattacks,
- Low power consumption during the authentication, which is very appealing to resource-constrained applications of the IoT.

The paper is organized as follows: in Section 2, we discuss three commonly used techniques in IoT device authentication and provide a literature review in Section 3. In Section 4, we present our proposed mutual authentication mechanism, followed by the protocol analysis in Section 5. The performance evaluation is provided in Section 6. We conclude our study of the proposed mechanism and present the next steps for future works in Section 7 and Section 8, respectively.

## 2. Authentication Techniques in IoT Devices

A secured mutual authentication is essential to ensure the privacy and confidentiality of the data exchange between a device and the server. Many protocols have been developed for authenticating IoT devices. The three main ones are: (1) the encryption/decryption cryptography authentication technique where an encryption/decryption algorithm is utilized for authentication, (2) the localization and device environmental data authentication technique where the placement and surrounding information of the IoT device can be used to authenticate it, and (3) the PUF authentication technique which makes use of the intrinsic characteristics of IC in the IoT device. We briefly discuss each technique in the subsections below.

### 2.1. Authentication via Encryption/Decryption

A device in the IoT system can be authenticated using either the symmetric or asymmetric key cryptography technique. In symmetric key authentication, the IoT device and the server share and store a secret key. The most popular algorithm used in symmetric key cryptography is the Advanced Encryption Standard (AES) [9]. In asymmetric key authentication, both the IoT device and the server use a public–private key pair, where the public key is shared but the private key is not shared between one other. The private key is used for signature generation, whereas the public key is used for signature verification [10]. Both symmetric and asymmetric methods need a key to be securely stored on the device. Various techniques have been developed for utilizing and protecting the secret cryptographic key in the device [11,12]. For example, there are credential specific software applications that can be configured with the purpose of managing the authentication credentials in a device [13]. Other techniques make use of physical components such as the Central Processing Units (CPUs) or ICs with memory storage and store the secret keys in their memory that is always power backed [14]. Storing the cryptographic keys on the IoT device has an intrinsic vulnerability to certain types of attacks, where the attacker either physically or remotely connects to the device and reveals the stored key, e.g., firmware attacks [5].

### 2.2. Authentication via Localization and Device Environmental Data

An IoT device can be authenticated by locating it or knowing information about its neighboring devices or a communication link’s characteristics. The server can collect the IoT device placement and the device surrounding environment information such as time of arrival and/or received signal strength [15], examine the collected information, and accordingly authenticate the IoT device. Localization of the device can be achieved by using different technologies such as Global Positioning System (GPS) or a base radio station as in the Wireless Local Area Network (WLAN) [16]. The smart phone also can be used in this technique. After the server registers the device identity information, location information, and authorization policies, the protocol performs the actual location-based authentication and authorization process every time the device requests access to some resources or service from the system [17].

### 2.3. Authentication via Physical Unclonable Functions (PUFs)

PUFs are a robust and lightweight solution to secure IoT devices [18] they are low cost and are resilient security resources [19]. PUF-based authentication protocols can perform authentication between an IoT device and a server. In this approach, the server sends a challenge *C* to the IoT device and evaluates the corresponding response *R* received from the device. The PUF is usually implemented in intrinsic IC and used for secure key generation and storage in security-related applications. Each PUF exploits the random variations in delays of wires and logic gates implemented inside the circuit [19]. These unique characteristics are obfuscated, obscure to the users and cannot be duplicated in a manufacturing process. Moreover, any execution of an invasive attack (hardware attack) to the circuit completely damages PUF, resulting in no useful information being extracted from the circuit. There are two levels of PUF architectures, namely, strong PUF and weak PUF in terms of implementations. The strength of the PUF depends on the number of unique Challenge–Response Pairs (CRPs) that a single device can generate [19,20].

#### 2.3.1. Weak PUFs

Weak PUFs support a relatively small number of CRPs, which make them more suitable for secure key storage and entity authentication techniques in IoT. The common types of weak PUF include the Ring Oscillator (RO) PUF and SRAM PUF [19]. Brief descriptions of these two types of weak PUFs are provided below.

- SRAM PUF

**Figure 1:**
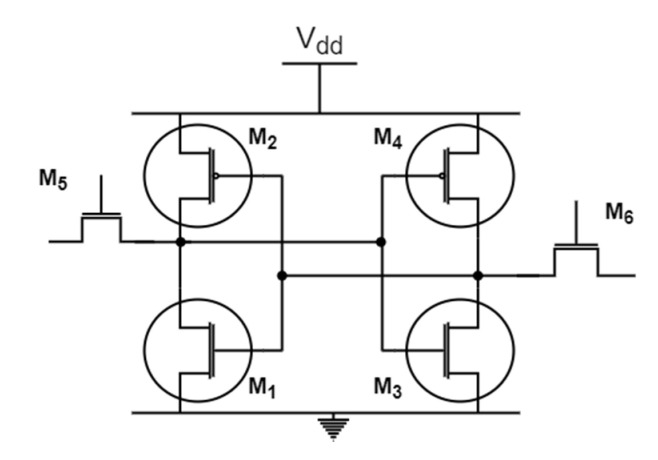
Model of SRAM cell.

Figure 1 shows the typical cell structure of a SRAM PUF. During the manufacturing process of the transistors small variations of electrical parameters will occur which results in threshold voltage mismatch between adjacent transistors. When the SRAM cell powers up, one of the two positive feedback loops will force the cell output to settle on a logic of “1” or “0” [19]. SRAM cell evaluation is based on the measurement of cell parameters such as the cell area, cell leakage, and the cell powers dissipated during the read and write operations. The measurements of these parameters can vary due to the silicon aging, electrical source noise, or temperature variations. Therefore, extracting a cryptographic key from SRAM PUF is not a straightforward process [21]. In the effort of extracting a reliable cryptographic key from the SRAM PUF with much less entropy, many algorithms have been proposed, which utilize fuzzy extractor and helper data as error correction methods to tackle the error probabilities of the SRAM cells values. An example of these methods can be found in [21,22] where authors were successfully able to extract stable SRAM output bits that can be used as a secret key (cryptographic key) to authenticate an IoT device without having to store this secret key in a non-volatile memory.

- Ring Oscillator (RO) PUF

Due to manufacturing variations, identically laid out delay loops produce oscillations with different frequencies. The dissimilarity of the frequency has been exploited to design the RO PUF [23]. RO PUF can be implemented on either an Application-Specific Integrated Circuit (ASIC), or a Field-Programmable Gate Array (FPGA) circuit. The design architecture of the typical RO PUF consists of *N* identical delay loops and counters. The oscillation frequencies are compared with each other in the given identical delay loops and the output value, one or zero, is equally likely to occur due to the random variations [19].

#### 2.3.2. Strong PUFs

Strong PUFs support a large number of CRPs in which a complete determination of the whole CRPs within a limited period of time is impractical [20]. A brief demonstration of the two types of strong PUFs are shown below.

- Arbiter PUF

**Figure 2:**
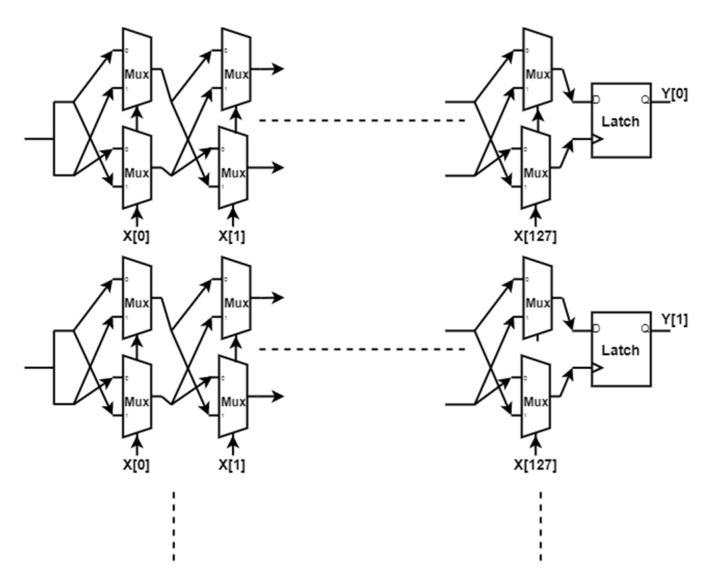
Model of 128-bit challenge Arbiter Physical Unclonable Function (PUF) circuit.

Gassend et al., in [24], described the silicon structure for strong PUF. The researchers showed that the gate circuit delay represents randomness that is feasibly unclonable. Figure 2 below shows one of the Arbiter PUF implementations in which two identical logic circuits are utilized to generate an output bit *Y* based on a race between the two circuits. The 128 input bits *X* [0] to *X* [127] represent the challenge and the output bit *Y* represents one bit of the PUF response *R*. The output bit *Y* changes according to the 128 input bits. A 128-bit output response is achieved by repeating the circuit in Figure 2 below 128 times to construct one Arbiter PUF circuit [19].

- Optical PUF

Optical PUF has been described by Pappu et al. [25] as a device that receives a laser input and outputs a corresponding pattern. Each output pattern (PUF response) is dependent on the challenge (PUF input) which is represented by the laser polarization and location *XY*.

## 3. Literature Review

Mughal et al., in [6], proposed an IoT authentication protocol based on PUF. In their authentication protocol, initially, the smart device registers its credentials, such as device serial number and fingerprint generated using PUF. Then, the gateway proves the authenticity of the device by matching the device PUF fingerprint with what is stored in the gateway’s repository. In case of a new device, the gateway registers its serial number after matching the device PUF fingerprint successfully. The smart device and the gateway share an initial secret key, the smart device stores the secret key in a non-volatile memory and the gateway stores the secret key in its repository. The initial secret key is then used to perform the initial message encryption and decryption processes. The proposed authentication scheme has to store an initial cryptographic key in the IoT device’s secure non-volatile memory to set up an initial connection with the gateway. That makes it vulnerable to being compromised by an attacker who has remote access to the IoT device and uses a firmware attack technique [5].

Aman et al., in [1], proposed a mutual authentication protocol based on PUF where the IoT device and the server authenticate each other. The proposed protocol constructs a session key using the hash of two random nonce values. When a new IoT device attempts the first access to the server, a password needs to be inserted into the device by a system operator, resulting in the device exchanging an initial CRP (*C^i^, R^i^*) with the server using the Time-Based One-Time Password (TOTP) approach [26]. The mutual authentication between the IoT device and the server is accomplished in three phases. First, the IoT device sends its identity, i.e., *ID_A_* and a nonce *N_1_* to the server. Second, the server tries to locate the CRP (*C^i^, R^i^*) that belongs to the received *ID_A_*, otherwise the communication will be rejected. After successfully locating the CRP in repository, the server sends *C^i^*, *M_A_*, and a Message Authentication Code (MAC(*M_A_*)) to the IoT device. The *M_A_* is a message of {*R_S1_*, *ID_A_*, *N_1_*} encrypted by the *R_i_* encryption key, and *R_S1_* is a randomly generated number by the server. Lastly, IoT device generates the response *R^i^* according to *C^i^*, obtains *R_S1_* by decrypting *M_A_* using *R^i^*, verifies the integrity of *M*_A_, and authenticates the source. If integrity or authentication fail, the IoT device terminates the communication. The session key in both the IoT device and the server is constructed using exclusive OR operation between Hash(*R_S1_*) and Hash(*N_A_*). Although their proposed algorithm is carefully designed to ensure message integrity, confidentiality, and freshness between the IoT device and the server, one of the unfavorable aspects is the aggregated power consumption in the encryption/decryption, hash, and MAC processes throughout the mutual authentication process. This is not desirable in resource-constrained systems.

Alizai et al., in [7], proposed a mutual authentication protocol that is based on public key encryption and the IoT device’s capability to calculate a predesigned functional operation. The functional operation can be a crypto puzzle or a function that can be calculated using the IoT device capabilities and a secret. Both the IoT device and the server store their own private key and the other’s public key. The proposed scheme for the device authentication is as follows: First, the IoT device sends a connection request to the server. Then, the server signs a nonce using its private key and sends the signed nonce alongside with a timestamp to the IoT device with the purpose of authenticating the IoT device. Second, the IoT device authenticates the server by verifying the server signature and the IoT device performs a functional operation on the received nonce, signs the output obtained from the functional operation using its private key, and sends the signed output alongside with a timestamp to the server. Lastly, the server decides whether the IoT device is authenticated by verifying the IoT device signature and the functional operation’s response. The proposed algorithm includes a timestamp to prevent a replay attack. The authors also mention that a window of five seconds will be used for the IoT device and the server to respond to a received request before the session timeout. There are two main drawbacks in this authentication protocol: Firstly, two secret keys have to be securely stored in a non-volatile memory in the IoT device which can be compromised and maliciously used by an attacker through firmware attack techniques. Second, using public key encryption in IoT mutual authentication is computationally expensive for resource-limited systems.

Han and Kim [8], proposed a lightweight mutual authentication scheme for IoT devices. In this scheme, a block cipher algorithm is deployed in the IoT device–server mutual authentication, where a secret key is initially stored in both the IoT device and the server, and a session key is dynamically generated using this secret key. The authentication mechanism is a typical application of using the encryption/decryption cryptography algorithms in authenticating IoT devices. As we can see, the proposed lightweight mechanism in [8] exhibits the same vulnerability as was previously explained about Mughal et al. and Alizai et al.’ protocols, where storing an initial secret in IoT devices can be compromised by an intruder using a firmware attack method.

Our literature review shows that the vulnerabilities of current authentication algorithms mainly stem from utilizing encryption techniques as in [6,7,8], where a secret key has to be stored in the IoT device’s non-volatile memory. Moreover, utilizing PUF, encryption/decryption, MAC, and hash algorithms all in one authentication scheme consumes relatively more power with respect to power-constrained IoT devices [1]. Therefore, we are proposing a new authentication mechanism for IoT devices that fits well for the resource-constrained devices and is resistant towards cryptanalysis attacks, as illustrated in the following sections.

## 4. Proposed Authentication Mechanism

In this section we provide a step-by-step description of the proposed mutual two-factor authentication mechanism between an IoT device and the server. Then, we list the steps for secure session key establishment for further data communication after a successful authentication.

### 4.1. Mutual Authentication Mechanism

Our proposed authentication algorithm is based on deploying two silicon PUFs in the IoT device, a strong PUF (Arbiter), and a weak PUF (SRAM), to achieve two-factor authentication. The mutual authentication is achieved by (1) the IoT device’s SRAM PUF secret key which *“only the server and the SRAM knows”* and (2) the IoT device’s Arbiter PUF, where the Arbiter PUF serves as the fingerprint of the IoT device which is “*something that characterizes the IoT device”*. Utilizing two PUFs in the IoT device provides a significant robustness throughout the mutual authentication process.

We use a keyed Hash-Based Message Authentication Code, in particular HMAC-SHA-256, in our proposed mechanism. HMAC uses a (one-way) hashing algorithm and supports verifying two significant security properties of a message: integrity and authenticity. It has high resistance against cryptanalysis and is considered a secure authentication technique [27]. Despite the fact that the SHA-3 hash standard algorithm has a proven security robustness and a high performance with a promising future in security applications, the migration from SHA-2 to SHA-3 has been slow. Therefore, we decided to consider using the SHA-2 hash standard algorithm in our proposed authentication mechanism as SHA-2 has been unbreakable since it was designed and published by the National Security Agency (NSA) in 2001. SHA-2 is implemented in well-known security applications such as Secure Sockets Layer (SSL) and Transport Layer Security (TLS). In addition, it has been utilized by several United States government applications to secure sensitive data. Moreover, SHA-2 has been implemented in many security ICs for commercial purposes. In contrast to SHA-3, SHA-2 has more hardware and software support which makes it easier and more trusted to utilize, whereas SHA-3 does not have the same level of support [28].

For application and transport layers, we assume utilization of the Constrained Application Protocol (CoAP), the common transfer protocol for power-constrained IoT devices. It is secured with the use of Datagram Transport Layer Security (DTLS) over User Datagram Protocol (UDP) [29]. The use of HMAC results in a lightweight authentication in terms of computational complexity as it utilizes only hash algorithms, which are fast compared to encryption/decryption algorithms, in particular, public key ones.

In the proposed mechanism, the server stores the identifier, SRAM’s intrinsic key, and Arbiter PUF’s Challenge–Response Pair (CRP) for each IoT device in its repository. These keys and CRPs are used when an IoT device wants to establish a connection with the server. To ensure message freshness and to prevent replay attacks, we send across a timestamp with each message throughout the authentication process.

**Figure 3:**
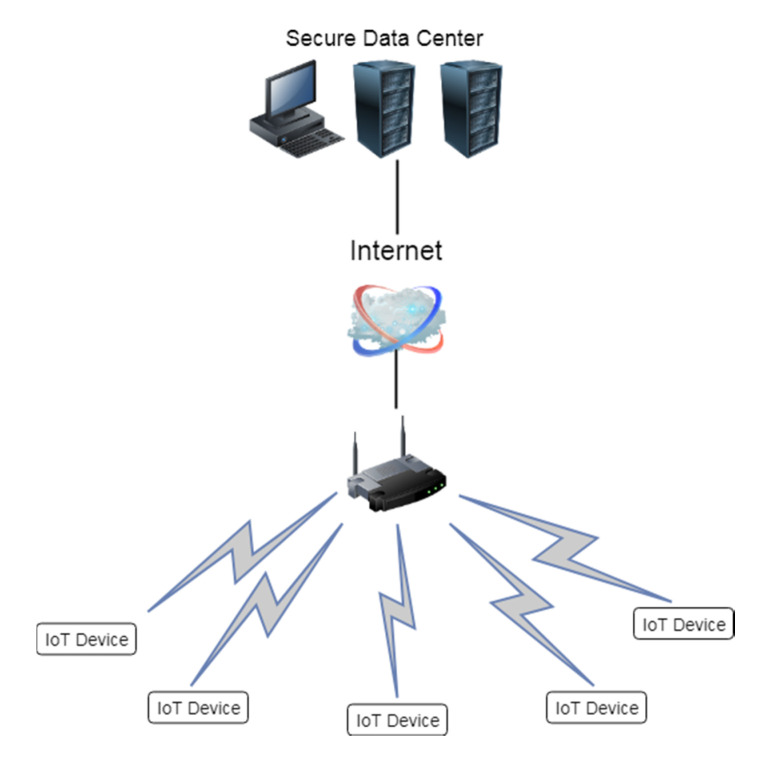
Internet of Things (IoT) system model.

**Figure 4:**
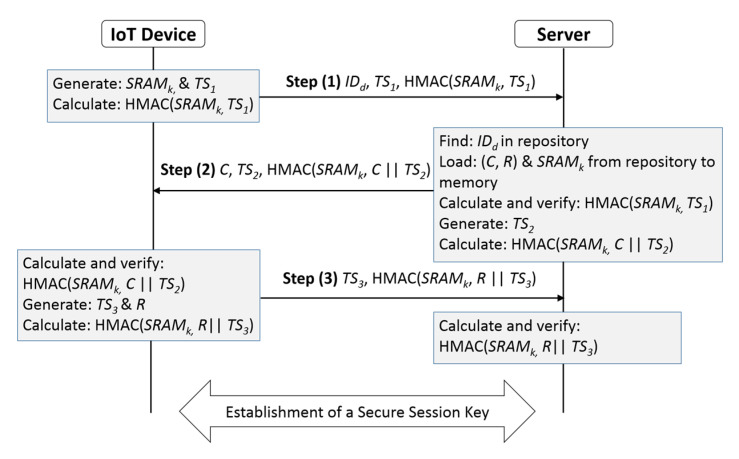
Proposed mutual authentication steps.

A symbolic model of the assumed system is shown in Figure 3. The IoT devices are assumed to communicate with the server through a public channel such as the Internet. Each individual IoT device is resource limited and is equipped with two PUFs. We assume that the server is in a highly secured environment in the data center. The notation used in the algorithm is described in Table 1. Figure 4 shows the steps in the mutual authentication protocol we propose. The details at each step are shown below.

In Step (1), the IoT device generates a cryptographic key *SRAM_k_* using its SRAM PUF, a timestamp *TS*_1_, and calculates a keyed hash message authentication code HMAC(*SRAM_k_, TS*_1_) using the HMAC-SHA-256 with the timestamp *TS*_1_ and the key *SRAM_k_*. Then, the IoT device sends its identifier *ID_d_*, the generated timestamp *TS*_1_, and HMAC (*SRAM_k_, TS*_1_) message to the server.

In Step (2), upon receiving the message from the IoT device, the server tries to locate the *ID_d_* of the IoT device in its repository. If it fails to locate it, the connection is rejected. Otherwise, the server finds the IoT device’s *ID_d_*, and loads the *SRAM_k_* that belongs to this *ID_d_* from its repository to its memory, then calculates the HMAC(*SRAM_k_, TS*_1_) message. The server matches both the received and the calculated hash messages to verify the IoT device’s message integrity. If the matching fails, the server terminates the connection. This verification process makes sure that the IoT device’s message was not tampered with or corrupted during transmission. This provides the first factor for authenticating the IoT device. The server then generates a timestamp *TS*_2_, copies the CRP (*C*, *R*) that belongs to the IoT device from its repository to its memory, generates (*C ‖ TS*_2_), and calculates HMAC(*SRAM_k_, C ‖ TS*_2_) message. The server then sends the challenge *C,* the timestamp *TS*_2_, and the HMAC(*SRAM_k_, C ‖ TS*_2_) message to the IoT device.

In Step (3), once the IoT device receives the server response, the IoT device generates (*C ‖ TS*_2_), and calculates HMAC(*SRAM_k_, C ‖ TS*_2_) message. The IoT device compares the received and the calculated hash messages. Because the server is the only device in the system that knows and stores the IoT device’s *SRAM_k_*, the IoT device will verify the authenticity of the server based on the success of matching the hash messages. If the message from the server to the IoT device was tampered with or corrupted during communication, then the matching process will fail and the IoT device will terminate the connection. Otherwise, the IoT device inputs the challenge *C* to its Arbiter PUF, and generates the corresponding response *R*. The IoT device then generates a timestamp *TS*_3_, generates (*R ‖ TS*_3_), and calculates HMAC(*SRAM_k_, R ‖ TS*_3_) message. The IoT device then sends the timestamp *TS*_3_, and HMAC(*SRAM_k_, R ‖ TS*_3_) message to the server.

Upon receiving the message from the IoT device, the server calculates HMAC(*SRAM_k_, R ‖ TS*_3_) and compares the received and the calculated hash messages. If they match, the server verifies the authenticity of the IoT device, as a successful matching of these two hash messages means that the server communicates with the right IoT device. However, if the matching fails, then the server terminates the connection. Note that this is the second factor used for authentication of the IoT device. In addition to utilizing a timestamp throughout the mutual authentication process, the protocol will utilize a time duration delta t (Δt), after which, a request originator (IoT device or server) will terminate the connection if it did not receive a response from the other party that it wants to establish a connection with. The time duration Δt can be determined by the system design engineer, which can be based on the IoT network architecture, and the IoT device surrounding environment. Maintaining time Δt during the authentication is a significant defense mechanism against a replay attack, where an attacker can try to delay the communication flow between the IoT device and the server. The time duration is not included in Figure 4 to keep it simple.

The algorithm for our proposed mutual authentication mechanism is shown in detailed steps in Algorithm 1.
**Algorithm 1** The mutual authentication between IoT device and server.**Input**: An IoT device with identity *ID_d_*, and server that stores the IoT device’s information (*SRAM_k_*, CRP, and *ID_d_*);**Output**: A mutual authentication between the IoT device and the server;**Begin**1: The IoT device generates a SRAM PUF cryptography key *SRAM_k_*, a timestamp *TS*_1_, and HMAC(*SRAM_k_*, *TS*_1_) message;2: IoT device sends its *ID_d_*, *TS*_1_, and HMAC(*SRAM_k_*, *TS*_1_) message to the server;3: **If** (the server finds *ID_d_* in its repository) **then**4:   The server loads the *SRAM_k_*, and CRP (*C*, *R*) that belongs to the *ID_d_* from its repository to its memory;5:   The server calculates HMAC(*SRAM_k_*, *TS*_1_) message;6:   **If** (the calculated hash message in step 5 matches the hash message that was sent in step 2)      **then**7:     The server generates a timestamp *TS*_2_, calculates (*C* ‖ *TS*_2_), and generates HMAC(*SRAM_k_*, *C* ‖ *TS*_2_) message;8:     The server sends *C*, *TS*_2_, and HMAC(*SRAM_k_*, *C* ‖ *TS*_2_) message to the IoT device;9:   **else**          Go to step 21;     **end if**10: **else**       Go to step 21;   **end if**11: The IoT device calculates (*C* ‖ *TS*_2_), and generates HMAC(*SRAM_k_*, *C* ‖ *TS*_2_) message;12: **If** (the calculated hash message in step 11 matches the hash message that was sent in step 8)     **then**       **The authenticity of the server is verified;**13:   The IoT device passes the challenge *C* to its Arbiter PUF, and generates a response *R*;14:   The IoT device generates a timestamp *TS*_3_, calculates (*R* ‖ *TS*_3_), and generates HMAC(*SRAM_k_*, *R* ‖ *TS*_3_) message;15: The IoT device sends *TS*_3_, and HMAC(*SRAM_k_*, *R* ‖ *TS*_3_) message to the server;16: **else**          Go to step 21;     **end if**17: The server calculates (*R* ‖ *TS*_3_), and generates HMAC(*SRAM_k_*, *R* ‖ *TS*_3_) message;18: **If** (the calculated hash message in step 17 matches the hash message that was sent in step 15)     **then**       **The authenticity of the IoT device is verified;**19:   A mutual authentication between the IoT device and the server is established;20: **else**         Go to step 21;      **then**21: **Stop** (terminates the connection);**End**

### 4.2. Secure Session Key Establishment

**Figure 5:**
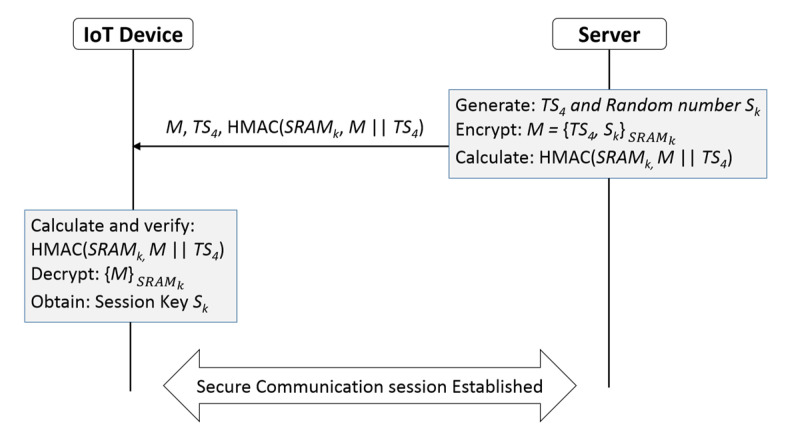
Establishment a secure session key.

In this subsection we propose a method for secure session key establishment between the server and the IoT device, with the assumption that they have achieved mutual authentication and trust each other. Figure 5 shows the main steps in secure key establishment. The details of the steps are shown below.

1. The server generates a 128-bit random number which will be the session key *S_k_* and timestamp (*TS*_4_) to ensure the freshness of the message.
2. The server encrypts {*S_k_*, *TS*_4_} using the IoT device cryptographic key *SRAM_k_* and a strong symmetric encryption algorithm such as the *AES* block cipher encryption algorithm (128-bit block size); *M* = $AES_{SRAM_{k}}\left(\left\{S_{k},\;TS_{4}\right\}\right)$.
3. The server generates HMAC (*SRAM_k_*, *M* ‖ *TS*_4_) and sends it alongside the message *M* and the timestamp *TS4* to the IoT device.
4. The IoT device generates (*M* ‖ *TS*_4_), and calculates HMAC (*SRAM_k_*, *M* ‖ *TS*_4_), and compares the received and the calculated hash messages for the purpose of verifying the message integrity. If the two hashes do not match, the IoT device terminates the connection. Otherwise, the IoT device decrypts the message *M* using its *SRAM_k_* and AES encryption algorithm and retrieves the secured session key *S_k_*.
5. The session key *S_k_* will be used by the IoT device and the server to encrypt and decrypt the exchanged data between the IoT device and the server.

If the communication session gets disrupted and the connection gets terminated for any reason, the IoT device and the server will have to repeat the entire mutual authentication steps that we described in Section 4.1 to re-establish a new session key.

We would like to highlight some features of our mechanism that distinguish it from other methods for mutual authentication between a server and IoT device: In the proposed mechanism, the Challenge–Response Pair (*C*, *R*) and the *SRAM_k_* for the IoT device are securely stored in the server’s repository. After the IoT device and the server create a session key *S_k_*, both the IoT device and the server delete the unneeded parameters from their memories. This includes the *TS*_1_, *TS*_2_, *TS*_3_, *TS*_4_, *C*, *R*, *ID_d_*, and *SRAM_k_*. No sensitive data are exchanged in plaintext or in encrypted form in our proposed mutual authentication method. Furthermore, the cryptographic key *SRAM_k_*, which is utilized for achieving mutual authentication and securing a session key, is securely stored only in a server located in a diligently protected data center. Therefore, even if an attacker intercepted the transferred messages between the IoT device and the server, they will not be able to reveal the cryptographic key *SRAM_k_* nor the session key *S_k_*. This supports the confidentiality of the session key and the security resilience of our proposed authentication mechanism.

## 5. Protocol Analysis

In this section, we provide brief descriptions of various types of attacks against IoT systems. Then, we describe how the proposed authentication mechanism stands strong against these common cyber threats due to the defense features included in its design.

### 5.1. Cyberattack Scenarios

A brief definition of the common cyber threats that an attacker can launch on an IoT system is demonstrated in Table 2.

Cyberattack scenarios may aim to compromise an initial encryption key which is used to encrypt/decrypt the exchanged authentication messages. They may also aim to compromise the session key which is usually used for data transfer between the IoT device and the server after successful mutual authentication.

### 5.2. Cyber Defense Assessment

Our proposed mutual authentication mechanism is strong against the cyberattack scenarios we have described in Table 2. The strength comes from deploying two different PUFs in the IoT device which allows no secret values or keys to be transmitted between the IoT device and the server. A defense assessment of the proposed scheme against the attack scenarios is given in the following points:

1. The IoT device and the server use timestamps in their communications which helps to prevent replay attack attempts.
2. Machine learning attack to predict the Arbiter PUF’s responses is not feasible in our mutual authentication. This type of attack is based on capturing PUF’s CRPs. In our mechanism, we do not exchange the PUF response in plaintext, we utilize only one pair of the Arbiter PUF’s CRP for each IoT device in the system. Therefore, launching a machine learning attack on our proposed protocol is not applicable (N/A). Machine learning or modeling attack requires capturing several CRPs of the PUF in order to train the model and predict the PUF response. Furthermore, if an attacker intercepted our exchanged PUF information, they will see only the PUF’s challenge which will be useless for the ML algorithms. The PUF’s CRP in our proposed scheme is similar to the user account’s password in any system, changing the user account’s password regularly is a good security practice. Similarly, the PUF’s CRP in our scheme can be changed to another CRP on a regular basis throughout the IoT device’s life cycle, which presents a strong security measure in the system. Note that changing the PUF’s CRP regularly does not require storing all the PUF’s CRPs in the production server. The PUF’s CRPs can be stored in backup storage for the future use.
3. In our authentication mechanism, the IoT device and the server do not exchange sensitive data (such as *SRAM_k_*, PUF response *R*, or the session key *S_k_*) in plaintext over the unsecure communication channel. This supports the data confidentiality. In fact, in the proposed scheme *SRAM_k_* and PUF response R are not exchanged in encrypted form either. No encryption takes place before the session key establishment. The only exchanged data in plaintext during authentication are the IoT device identity *ID_d_*, timestamps (*TS*_1_, *TS*_2_, *TS*_3_), PUF’s challenge *C*, and hashed messages. In such a setting a man-in-the-middle attack will not benefit from any captured data. Furthermore, if the man in the middle alters the transferred data, the intended receiver will detect the alteration by matching the hashed messages and drop the connection.
4. A silicon PUF cannot be duplicated due to its unique characteristics [19]. An invasive attack on the IoT device PUF to discover its structure will not succeed as duplicating the discovered structure is infeasible. Because the initial secret key is not stored on a chip in the IoT device, there is no useful information on the IoT device for an invasive attack technique to benefit from.
5. As we are utilizing a SRAM PUF in our authentication scheme, we do not store the secret key in memory in the IoT device. Therefore, launching a firmware attack on the IoT device will fail to reveal any useful information.
6. In our proposed authentication mechanism, although we do not exchange the Arbiter PUF response with any device, it is possible that a side channel attack could succeed in recovering the Arbiter PUF response. However, in our authentication mechanism, revealing the Arbiter response will be useless because: (1) we do not use the Arbiter response to generate the session key and (2) there are no sensitive data that can be recovered by knowing the Arbiter response. Therefore, an attacker would have to launch a successful side channel attack on the SRAM PUF to recover its key *SRAM_K_* to compromise the session key. It is possible to have the SRAM PUF key highly secured as there have been attempts in research for improving the security level of the SRAM PUF. One successful study in this field is presented in [32], where researchers present a highly efficient and secure mechanism for extracting and securing the SRAM PUF key against side channel attacks. Such a solution increases the reliability of using SRAM PUF in IoT device security systems. Our approach of utilizing two PUFs in the IoT device and server authentication reduces the effectiveness of side channel attacks against the authentication of the devices, compared to authentication mechanisms that deploy one PUF.
7. Assume that an attacker somehow gets hold of the SRAM key *SRAM_k_* of the IoT device. To launch a successful spoofing attack, they need to also know the Arbiter PUF responses. Since we do not exchange the PUF response *R* in plaintext and the Arbiter PUF practically cannot be duplicated, a spoofing attack against our mutual authentication mechanism is impractical.
8. As we mentioned above in point number 3, our mutual authentication mechanism does not exchange sensitive data in plaintext. Therefore, if an attacker sniffs out the communication link between the IoT device and the server, they will fail to reveal any useful information that might help to obtain any sensitive data.

In Table 3, we provide a comparison of our proposed mutual authentication mechanism and other recent PUF-based authentication schemes. Our proposed scheme is the only one that resists all the considered attacks. In particular, apart from Aman et al.’s authentication scheme, our scheme is the only one that resists against firmware attacks which aim at compromising the secret keys fuzzy in non-volatile memory.

The consequences of compromising the secret key stored in the memory would be devastating. The attacker could exploit the compromised key to initiate different types of attacks, as in [6,7,8], and possibly reconstruct the session key. This would enable the attacker to compromise sensitive data and use them in malicious activities. However, compromising the secret key and/or trying to reconstruct the session key is not possible in our proposed mutual authentication mechanism.

### 5.3. Formal Analysis

In this section, we conduct a type of formal security analysis to validate the protocol’s security and prove its trustworthiness as a mutual authentication protocol for IoT devices. Our formal analysis is based on the Burrows, Abadi, and Needham (BAN) belief logic of authentication [34]. The BAN logic can be used as an illustrative method of the essential concepts of an authentication protocol, and as a basic verification tool for a security protocol.

#### 5.3.1. BAN Logic Assumption and Postulates

The BAN method formalizes the authentication protocol by symbolically representing the protocol’s objects, i.e., the communication ends are called principals, and can be represented by *A*, *B*, and *S*. Other protocol objects such as exchanged messages and shared secret keys take different symbols. The protocol validation in BAN logic is inferred by applying some sets of assumptions and logic postulates. The set of BAN assumptions and logical postulates that are used in the formal analysis of our proposed mutual authentication protocol are as follows:

##### Assumptions

- *P*| ≡ *X*: Principal *P* believes that *X* is true. Principal *P* represents the IoT device or the server in our protocol, whereas *X* represents an exchanged message between the IoT device and the server.
- *P* ⊲ *X*: Principal *P* sees, can read and repeat *X.*
- *P*|~ *X*: Principal *P* believed *X* and sent it once.
- *P*⟹*X*: Principal *P* has the authority to generate *X*, and *P* should be trusted for that.
- ♯(*X*): Represents the freshness of the message *X*.
- *P* $\overset{K}{↔}$*Q*: The key *K* is good for the communication between the principals *P* and *Q*, where no one can compromise the key *K*.
- *P* $\overset{X}{\Leftrightarrow }$*Q*: The message *X* is a shared secret, which is known only to the principals *P*, *Q*, and any other principals that are trusted by them.
- ${\left(X\right)}_{Y}$: This represents *X* and *Y* as a compound, where *Y* is a secret, and the presence of *Y* proves the identity of the principal that sent ${\left(X\right)}_{Y}$.

##### Logical Postulates

Here, we represent the postulates as set of rules, on which our analysis will be based.

Rule (1) is the message-meaning rule: It means that if principal *P* believes that *K* is a good shared secret key with principal *Q*, and *P* sees the message *X* which is encrypted by *K*, then principal *P* believes that principal *Q* once has said the message *X.*

(1) $$\frac{P|\equiv \;Q\overset{K}{↔}P,\;P\;⊲\;{\left\{X\right\}}_{K}\;}{P\left|\equiv \;Q\right|\sim \;X}$$

Rule (2) is the shared secret rule: It means that if principal *P* believes that *Y* is a secret shared with principal *Q*, and *P* sees the message *X*, then principal *P* believes that principal *Q* once has said the message *X.*

(2) $$\frac{P|\equiv \;Q\overset{Y}{\Leftrightarrow }P,\;P\;⊲\;{\left(X\right)}_{Y}\;}{P\left|\equiv \;Q\right|\sim \;X}$$

Rule (3) is the nonce verification rule: It means that if principal *P* believes that *X* “which should be a plain text” has been recently generated, and that principal *Q* has said *X* once, then principal *P* believes that principal *Q* believes *X.*

(3) $$\frac{P|\equiv \;♯\left(X\right),\;P\left|\equiv \;Q\right|\sim \;X}{P\left|\equiv \;Q\right|\equiv \;X}$$

Rule (4) is the jurisdiction rule: It means that if principal *P* believes in the jurisdiction of principal *Q* over *X*, then principal *Q* guarantees the truth of *X* and principal *P* trusts principal *Q* on that.

(4) $$\frac{\;P\left|\equiv \;Q⟹X,\;P\right|\equiv \;Q|\equiv \;X}{P|\equiv \;X}$$

Rule (5) is the belief rule: It means that principal *P* believes a combination of statements, only if *P* believes each single statement of that combination.

(5) $$\frac{\;P|\equiv \;\left(X,\;Y\right)}{P|\equiv \;X}$$

Rule (6) is also a belief rule: It means that principal *P* believes that principal *Q* said a combination of statements, only if *P* believes that *Q* has said each single statement of that combination.

(6) $$\frac{P\left|\equiv \;Q\right|\sim \;\left(X,\;Y\right)}{P\left|\equiv \;Q\right|\sim \;X}$$

Rule (7) is another belief rule: It means that principal *P* believes that it sees a combination of statements, only if *P* believes that it sees each single statement of that combination.

(7) $$\frac{\;P⊲\left(X,\;Y\right)}{P\;⊲\;X}$$

Rule (8) is freshness rule: It means that if principal *P* believes that the message *X* is fresh, then *P* must believe that ${\left(X\right)}_{Y}$ is also fresh, the same belief holds true if it was ${\left\{X\right\}}_{K}$ in the denominator instead.

(8) $$\frac{\;P|\equiv \;♯\left(X\right)}{P|\equiv \;♯{\left(X\right)}_{Y}}$$

Rule (9) is the hash function *H* rule: It means that if principal *P* believes that principal *Q* said a hashed composite message, and *P* could see each individual component of the composite message, then principal *P* believes that principal *Q* could say each individual component of the composite message once.

(9) $$\frac{\;P\left|\equiv \;Q\right|\sim \;H\left(X_{1},\ldots ,X_{k}\right),\;P\;⊲\;X_{1},\ldots ,\;P\;⊲\;X_{k}}{P\left|\equiv \;Q\right|\sim \;(\left(X_{1}\ldots X_{k}\right)}$$

#### 5.3.2. Proposed protocol Detailed Analysis

Based on BAN logic methodology, we are going to represent our authentication protocol in its idealized form, which represents the protocol message, after omitting the message’s parts that have no effect on the recipient’s belief of the message, i.e., the plain text in the message. Hence, the idealized protocol is represented as follows:

For the simplicity, we notate the IoT device by ‘D’, and the server by ‘S’ in our analysis:

In step 1: *D* ⟶ *S*: *H*(${\left(X_{1}\right)}_{SRAM_{k}}$), where *X*_1_ is *TS*_1_

In step 2: *S* ⟶ *D*: *H*(${\left(X_{2}\right)}_{SRAM_{k}}$), where *X*_2_ is *C* ∥ *TS*_2_

In step 3: *D* ⟶ *S*: *H*(${\left(X_{3}\right)}_{SRAM_{k}}$), where *X*_3_ is *R* ∥ *TS*_3_

The in-session key generation step is:

*S*⟶*D*: *H*(${\left(X_{4}\right)}_{SRAM_{k}}$), where *X*_4_ is (${\left\{TS_{4},\;S_{k}\right\}}_{SRAM_{k}}$ ∥ *TS*_4_)

Here, we state a set of assumptions for our proposed protocol:

*D*|≡ *D* $\overset{SRAM_{k}}{↔}$ *S*, *S*|≡ *D* $\overset{SRAM_{k}}{↔}$ *S*: Both assumptions indicate that the IoT device and the server initially share a secret key, which is the SRAM secret key *SRAM_k_*.

*D*|≡ (*S* ⟹ *D* $\overset{K}{↔}$ *S*), *S*|≡ *D* $\overset{S_{k}}{↔}$ *S*: Both assumptions indicate that the server has generated a new session key *S_k_*, and that the IoT device trusts the server to generate good session keys.

*D*|≡ ♯(*TS*_1_), *D*|≡ ♯(*TS*_3_), *S*|≡ ♯(*TS*_2_), *S*|≡ ♯(*TS*_4_): These assumptions indicate that the IoT device and the server are able to generate a fresh nonce.

*D*|≡ *D* $\overset{R}{\Leftrightarrow }$ *S*, *S*|≡ *D* $\overset{R}{\Leftrightarrow }$ *S*: Both assumptions indicate that the IoT device and the server are sharing a secret statement, which is the Arbiter PUF response *R* to the challenge *C* during the mutual authentication process.

Now, from step 1 in the idealized protocol that mentioned above, and rules (2, 3, 8, 9), we can simply deduce that *S*|≡ *D*|~ *TS*_1_, *S* ⊲ *TS*_1_, which means that the server believes that the IoT device has said *TS*_1_, and the server can see *TS*_1_. As from the assumptions, we have that the IoT device guarantees the freshness of *TS*_1_, and the IoT device and the server initially share the secret key *SRAM_k_*. Thereby, the server here will have a partial belief in the existence of the IoT device; however, at step 3, the server will be highly certain of the existence of the IoT device, as we will illustrate later. From step 2 in the idealized protocol, and rules (2, 3, 5, 6, 7, 8, 9) we can deduce that *D*|≡ *S*|~ (*C* ∥ *TS*_2_), *D* ⊲ *C*, and *D* ⊲ *TS*_2_, which means that the IoT device believes that the server has said the composite statement (*C* ∥ *TS*_2_), and the IoT device can see the challenge *C* and *TS*_2_. Similarly, from our assumptions, we can see that the server guarantees the freshness of *TS*_2_, and the IoT device and the server know that they initially share the secret key *SRAM_k_*. Thus, here, the IoT device is certainly assured of the existence of the server. From step 3 in the idealized protocol, and rules (2, 3, 5, 6, 7, 8, 9) we can deduce that *S*|≡ *D*|~ (*R* ∥ *TS*_3_), *S* ⊲ *R*, and *S* ⊲ *TS*_3_, which means that the server believes the IoT device has said the composite statement (*R* ∥ *TS*_3_), and the server can see the IoT device’s Arbiter PUF response *R* and *TS*_3_. As we can see from our assumptions, the IoT device guarantees the freshness of *TS*_3_, the IoT device and the server share the secret key *SRAM*_k_ as well as the Arbiter PUF secret response *R*. Therefore, at this point, the server is certainly assured of the existence of the IoT device.

Hence, the server and the IoT device have mutually authenticated each other. Now the server can send a new session key to the IoT device. This can be shown in the session key generation step from the idealized protocol. As we can see from our assumptions, the IoT device trusts the server on generating good new session key *S_k_*, applying rule (4) states that the IoT device believes the authority of the server to generate good new session keys, the server guarantees the freshness of *TS*_4_, and the IoT device and the server share the secret key *SRAM_k_*. Therefore, we can infer the final protocol beliefs:

*D*|≡ *S*|~ (*D* $\overset{S_{k}}{↔}$ *S*, *TS*_4_): The IoT device believes that the server has said a good new session key *S*_k_.

*S*|≡ *D* $\overset{S_{k}}{↔}$ *S*: The server believes that it shares a good new session key with the IoT device.

*D*|≡ *S*|≡ *D* $\overset{S_{k}}{↔}$ *S*: The IoT device believes that the server guarantees the goodness of the session key *S*_k_.

*S*|≡ *D*|≡ (*TS*_2_, *TS*_4_): The server knows that the IoT device believe the freshness of the two timestamps *TS*_2_, and *TS*_4_.

*D*|≡ *S*|≡ (*TS*_1_, *TS*_3_): The IoT device knows that the server believes the freshness of the two timestamps *TS*_1_, and *TS*_3_.

Thus, by stating the above final beliefs, we validate our proposed mutual authentication protocol. We formally prove our protocol correctness as a secure mechanism to authenticate an IoT device and server, and to distribute a new session key that can be used for further data communication between the IoT device and the server.

## 6. Performance Evaluation

In this section, we analyze and evaluate the performance of our proposed authentication mechanism based on the computational complexity, communication overhead, and storage constraints. We compare our mutual authentication mechanism to one of the most recent and relevant PUF-based mutual authentication mechanism proposed by Mughal et al. [6].

### 6.1. Computational Complexity

The main operations in PUF-based IoT authentication schemes are hashing, encryption, and PUF operations. Table 4 below shows the time required in the IoT device and server for authentication in the proposed scheme and in Mughal’s scheme. The time is represented in terms of the main operations required. The symbol *N_H_* represents the time for hashing, *N_PUF_* represents the time for PUF operations, *N_ENC_* represents the time for encryption/decryption, and *N_HMAC_* represents the time for computing a hashed message authentication code.

**Figure 6:**
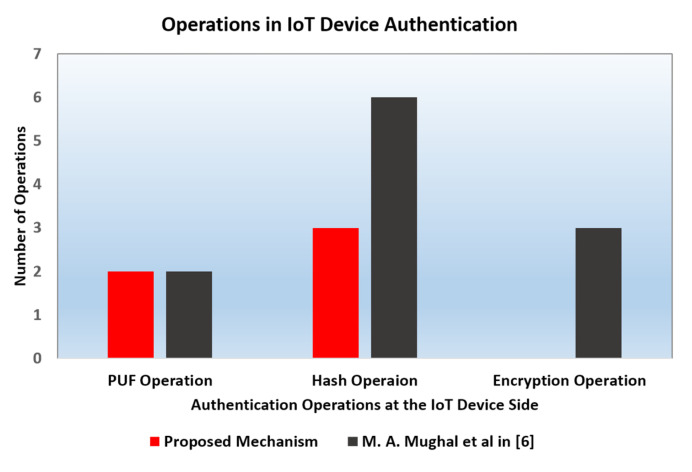
Comparison of the number of operations in IoT device authentication of the proposed mechanism and Mughal et al.’s mechanism.

We assume the use of the AES block cipher encryption algorithm. The block size for AES is 128 bits and the key size can be one of 128, 192, or 256 bits [35]. The average encryption/decryption times vary based on the length of the AES key length. The encryption/decryption takes longer time and more processing power with the increase in the AES key length [36]. In Figure 6, the number of operations in the IoT device authentication of our proposed mechanism is compared with that of the mutual authentication mechanism in [6]. We assume that the computational complexity of the *N_H_* operation is approximately equal to that of the *N_HMAC_* operation. As depicted in Figure 6, both mechanisms have the same number of PUF operations. On the other hand, the hash operation number of Mughal’s algorithm is double our proposed authentication protocol’s. Note that our proposed authentication protocol uses only hashing without encryption operation and has a lower computational complexity than Mughal’s protocol.

### 6.2. Communication Overhead

In order to compare the communication overhead of our mutual authentication mechanism with that of Mughal et al., we list the message parameters that are communicated between the server and the IoT device along with their sizes in Table 5 (The numbers in brackets are references). Based on the values in Table 5 and the protocol steps for authentication and session key establishment in Figure 4 and Figure 5 respectively, we infer that the total number of the transmitted bytes between the IoT device and the server is 201 bytes. Which represents the communication overhead of our proposed protocol for establishing secure data communication between the IoT device and the server.

**Figure 7:**
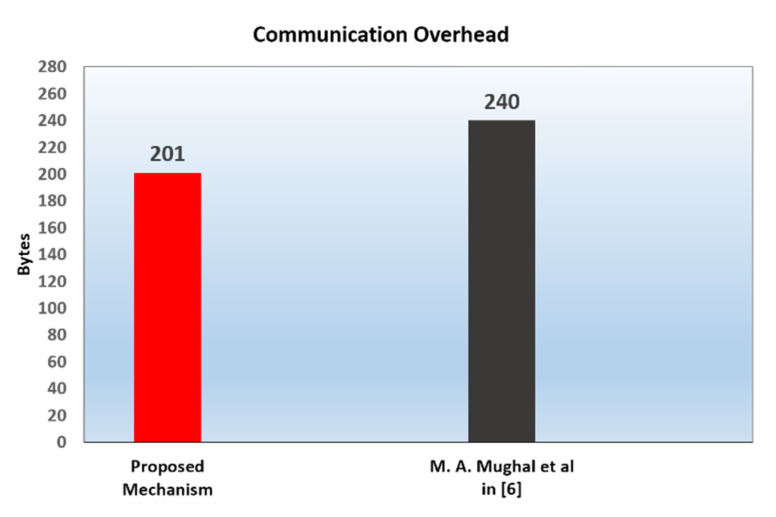
Comparison of communication overhead of the proposed scheme with Mughal et al.’s scheme.

Assuming the use of the AES block cipher encryption algorithm (128-bit block size) in Mughal et al. protocol with the sizes given in Table 5 for the messages’ parameters, the communication overhead of Mughal et al.’s protocol is 240 bytes. Again, this is the total number of the transmitted bytes between the IoT device and the server for establishing secure data communication in their scheme. Comparing the two protocols, the communication overhead in our proposed mutual authentication mechanism is lower than the Mughal et al. mechanism by 16.25%, as represented in Figure 7.

### 6.3. Storage Constraints

Our proposed mutual authentication mechanism does not impose any constraints on the IoT device’s storage for storing any sensitive data, as the IoT device does not store the secret key. Moreover, after the establishment of secure connection, the IoT device and the server delete all random numbers that have been used during the authentication process. The server stores only an identifier and a CRP (*C*, *R*) for each IoT device in the system. Therefore, the server in our proposed authentication mechanism is more scalable for a large number of IoT devices that could be deployed in the system. On the contrary, in [6] IoT device has a storage constraint as it has to store an initial secret key in its memory. The server stores the IoT device identifier, the IoT device serial number, the IoT device fingerprint, and the IoT device PUF CRPs. Hence, with a large number of IoT devices in the system, a large server storage size will be required, which presents a server un-scalability issue.

## 7. Conclusions

In this study, we proposed a novel mutual two-factor authentication protocol between a server and an IoT device that requires the use of only hash functions We also provided a mechanism for secure session key establishment. We presented the concept of the proposed protocol in terms of cyberattack assessment, formal security analysis, and computational complexity. Our careful analysis validated the correctness of the proposed authentication protocol and our cyber defense assessment showed that it is infeasible to compromise the protocol even if an adversary conducted an invasive attack against the IoT device. The proposed authentication mechanism is the first mechanism that replaces the traditional use of encryption for authentication by HMAC computation. It deploys two PUFs in the IoT device to offer the desired level of security. It also achieves the required low computational complexity for power-constrained IoT applications.

## 8. Future Work

In this paper, we provided the main concept of a PUF and hash based mutual two-factor authentication scheme for IoT devices. We analyzed the scheme on the assumption that the robust and secure SHA-2 algorithm is used for the hash algorithm. Other hash functions can be used instead. We plan to expand this work to analyze the use of block cipher-based hash functions especially when the confidentiality of the transmitted data is desired. We also plan to develop a proof-of-concept and analyze the practical efficiency and performance from a hardware point of view and security in various attack scenarios, especially when combinations of attacks are launched against the system at the same time. In addition, we plan to assess the resilience of the SRAM key generation against Denial of Service (DoS) attacks.

## Figures and Tables

**Table 1:** Algorithm botation description.

Notation	Description
‖	Concatenation symbol
*ID_d_*	IoT device’s identifier
*TS*_1_, *TS*_2_, *TS*_3_, and *TS*_4_	Timestamps
*SRAM_k_*	Secret key (identifier) extracted from SRAM
*HMAC(SRAM_k,_ TS*_1_ *)*	A keyed hash message authentication code of the timestamp *TS*_1_
*C*	Challenge for the Arbiter PUF of the IoT device
*R*	Arbiter PUF’s response for the *C* challenge
*CRP (C, R)*	Challenge–Response Pair of *C* and *R*
$AES_{SRAM_{k}}\left(\left\{S_{k},\;TS_{4}\right\}\right)$	Encrypting $\left\{S_{k},\;TS_{4}\right\}$ using AES algorithm and the *SRAM_k_* as the cryptography key

**Table 2:** Cyberattack scenario descriptions.

ID.	Attack Scenario: Definition
01	**Replay attack**: A type of network attack where a legitimate traffic is maliciously repeated or delayed in order to disrupt the communications.
02	**Machine Learning (ML) attack**: PUF’s CRPs can be exploited to predict the PUF response by using ML algorithms.
03	**Man-in-the-middle attack**: A type of attack where an attacker secretly intercepts the traffic between two parties and possibly alters the communicated information.
04	**Invasive attack**: It is a hardware attack, where an attacker physically accesses the hardware’s semiconductor, i.e., an IC, with the purpose of reading the stored secrets on it or discovering its structure [30].
05	**Firmware attack**: An attacker remotely connects to the IoT device and alters or injects a malicious software to compromise the stored secret key.
06	**Impersonating/spoofing attacks**: A type of attack where an attacker tries to act as if they are a trusted node in an IoT system.
07	**Eavesdropping attack**: A form of sniffing attack where an attacker seeks out sensitive data from insecure communication networks.
08	**Side channel attack**: A type of attack which requires physical access to the PUF. The attack can be invasive, semi-invasive, and non-invasive. In the first two types, the attacker will have access to the internal structure of the PUF. The attack would not damage the PUF if semi-invasive, but it could damage the PUF if invasive. In non-invasive attack, the attacker would try to recover PUF secret key by analyzing data such as the PUF power consumption. Side channel attacks can be active where the attacker actively manipulates the PUF environment such as its power supply, or they can be passive, where the attacker is only collecting data about the PUF, such as the temperature, and analyzes these data [31].

**Table 3:** Comparison of the cyber defense capabilities of our proposed mutual authentication mechanism and the most recent PUF-based mutual authentication methods.

Security Feature	Mughal et al. [6]	Aman et al. [1]	Alizai et al. [7]	Han and Kim [8]	Xu et al. [32]	Banerjee et al. [33]	Proposed Mechanism
Utilized security algorithms	PUF and Encryption and Hash algorithms	PUF and Encryption and Hash algorithms	Functional Operation and Encryption and Hash	Encryption (Block Cipher algorithm)	PUF and Encryption	PUF and Hash algorithm and XOR operation	**PUF and Hash algorithm**
Number of security methods used	3	3	3	1	2	3	2
Two-Factor Authentication	Yes	No	Yes	No	No	Yes	Yes
Resilience to replay attack	No	No	Yes	No	Yes	Yes	Yes
Resilience to machine learning attack	No	Yes	No	N/A	N/A	N/A	N/A
Resilience to Man–in-the-middle attack	No	No	No	No	Yes	Yes	Yes
Resilience to invasive attack	Yes	Yes	N/A	N/A	Yes	Yes	Yes
Resilience to firmware attack	No	Yes	No	No	No	No	Yes
Resilience to spoofing attack	No	Yes	No	No	Yes	Yes	Yes
Resilience to eavesdropping attack	No	Yes	No	No	Yes	Yes	Yes

**Table 4:** Computational complexity comparison between the proposed mechanism and the Mughal et al.’s authentication mechanism.

Authentication Mechanism	IoT Device	Server
**Mughal et al. [6]**	6*N_H_* + 2*N_PUF_* + 3*N_ENC_*	6*N_H_* + 3*N_ENC_*
**Proposed Mechanism**	2*N_PUF_* + 3*N_HMAC_*	3*N_HMAC_*

**Table 5:** Authentication messages’ parameter values.

Message Parameters	Size in Bits
IoT device/Server ID [37]	8
Nonces [38]	64
CRP (*C*, *R*) [19]	128
HMAC o/p [39]	256
Hash function o/p [39]	256
Timestamps *TS*_1_, *TS*_2_, and *TS*_3_	Assumed 48
IoT Device Serial No.	Assumed 64
